# James Van Zandt Blaney, A. M., M. D.

**Published:** 1875-01

**Authors:** 


*At 1 o'clock A. M., on Friday, December 11th,*

1874,

### JAMES VAN ZANDT BLANEY, A.M., M.D.,

FOUNDER AND FIRST EDITOR

— OF THE —

CHICAGO MEDICAL JOURNAL.

---

"*Nec Prosunt Domino Quæ Prosunt Omnibus Artes.*"

THE

### Chicago Medical Journal.

A MONTHLY RECORD OF

*Medicine, Surgery and the Collateral Sciences.*

EDITED BY J. ADAMS ALLEN, M.D., LL.D.: AND WALTER HAY, M.D.

VOL. XXXII. — JANUARY, 1875. — NO. 1.

### Obituary.

JAMES VAN ZANDT BLANEY expired on the 11th of December, inst., at one o'clock in the morning, being in his 55th year.

His death is a shock to the scientific world, and a deep affliction to a wide circle of intimates and friends. The pulpit, the press and the public have united to do him honor, and have laid their tribute on his tomb. After a life of distinguished service to truth and humanity he sleeps well.

Dr. Blaney was born May 1st, 1820, at New Castle, Delaware. He sprang from a good stock. His parents were gentlefolks, and their home was the resort of the distinguished men of that day. John M. Clayton, Judge Baldwin, and Dr. McClellan, were frequent guests. The then youthful subject of this sketch was a delighted listener to their animated and polished conversation. His native love of truth and keen insight were quickened by association, and he was moved to a noble ambition.

He excelled in boyish sports, and keenly enjoyed them, but his thirst for knowledge was insatiable. While others were idle, he listened, and thought, and studied. He learned everything readily, and laid up great stores; but he preferred natural science, and devoted himself to it. He graduated at Princeton when only 18 years old, but remained there some months as a resident graduate, pursuing his studies under the distinguished Prof. Joseph Henry. He had already become a learned and skillful chemist, and had taken a survey of the kindred sciences.

From Princeton he went to Philadelphia and studied medicine under the great masters there. He completed his course before he arrived at age, and perfected himself by practice in the hospitals. He also pursued his favorite science of chemistry in connection with Prof. Booth, afterwards director of the mint.

Early in the year 1843 he set out for the West, armed with letters to leading men in this region. He visited the Mammoth Cave, was a guest at Ashland, was stationed for a season at Jefferson Barracks, sailed up the Mississippi to St. Paul, and finally, in the fall of the year 1843, arrived at Chicago.

His work here was not done in a corner. He associated with the late Dr. Daniel Brainard in founding Rush Medical College. His first chair united Chemistry, Pharmacy and *Materia Medica*, and the young man of 23 years proved an acceptable lecturer on all these topics. At the same time he engaged in the practice of medicine with ardor and success. His enterprises prospered, and his sphere of effort widened. He soon became a popular lecturer on scientific subjects, and delivered many courses of lectures before the Mechanics' Institute and other associations. He was an efficient member of the Board of Education, and was instrumental in building Dearborn schoolhouse, the first permanent school building in the city.

In May, 1846, he organized an expedition to explore the southern shore of Lake Superior. He was accompanied by the late Dr. J. H. Bird, who acted as civil engineer. The result was the discovery of mineral deposits, which have since been mined to great profit.

Dr. Blaney was in active practice during all the cholera years, devoting his great skill to rich and poor alike. Night and day he pursued his errand of mercy, bringing healing to many.

He was the founder of the CHICAGO MEDICAL JOURNAL, and for many years its Editor. His treasures of knowledge were poured out to enrich its pages, and his name and influence aided in giving it a wide circulation. His services as an analytical chemist were constantly in request. His aid and opinion were sought by an army of inventors, miners, manufacturers, dealers. As a detector of poisons in criminal cases, he became "a terror to evil doers, and a praise to them who did well."

The reader will remember the celebrated case of George W. Green. He was tried in this city on the charge of poisoning his wife, and was convicted on the testimony of Dr. Blaney. By the use of novel tests the Doctor found strychnine in the stomach of the murdered woman, and testified to it. He was critically cross-examined, but sustained himself. His analysis was published on both sides of the Atlantic, and was at first the subject of some adverse criticism. But he defended himself successfully, and won the assent of the scientific world. The criminal himself said to him: "Dr. Blaney, God guided your investigations!" and the same night the wretched man hung himself in his cell.

In a less conspicuous case of the same sort Dr. Blaney was the means of saving life. An expert testified to the presence of arsenic in the stomach. Dr. Blaney was called for the defense, and overwhelmed the prosecution. After denying the presence of arsenic, he turned to the presiding Judge and said: "Your Honor, if the test of the gentleman is to be relied on, I can prove the presence of arsenic in the water your Honor is drinking." And he made good his point by applying the test to the water on the spot.

In labors like these, Dr. Blaney employed himself until about the year 1857. By this time his reputation had become national, and his name was known beyond the seas. He had received and declined many invitations to other fields of honor and influence. But, being invited to the chair of Chemistry and Natural Philosophy in the Northwestern University, at Evanston, he accepted, and removed with his family to that place. He was impelled to this step by the conviction that he could not with safety continue the general practice of medicine, and by his love of country life. He fitted up a beautiful home, and laid out on a sand ridge a garden marvelous for its floral display, and for the variety and excellence of its fruits and vegetables.

At the breaking out of the war of the Rebellion he was summoned to Washington and entered the army as Surgeon, with rank of Major of Cavalry. Here his high qualities secured him places of trust. He was for a year Medical Director of the Department of Virginia and North Carolina with head quarters at Norfolk, and was with General Sheridan at the battle of Winchester, having charge of the hospitals. He remained in the army until the close of the war, serving with zeal and winning promotion. Under his administration ample provision was made for the sick and wounded, and no emergency found him unprepared. Upon the return of peace he resumed the duties which had been partially interrupted. He was solicited to practice as a physician, and consented to do an office business, and was called in consultation in difficult cases. He never resigned his chair in Rush Medical College, and he now resumed his regular courses of lectures. He was again an analytical chemist, and an authority in science. Upon the death of Dr. Brainard in the fall of 1866, he succeeded to the presidency of Rush Medical College, and wearing the harness while he had strength to endure, he pursued his career to the end.

In forming Dr. Blaney, nature was bountiful. He was above the middle height, of a lithe and graceful figure, crowned by a noble, well poised head, and a face full of thought and feeling—a sensitive and kindly face, full of dignity and courtesy. In mind he was gifted beyond the common lot. His powers were disciplined and always in the field. A new thought found its appropriate place and was never in the way. He loved science and pursued it, and made it his own. He found—

> "tongues in trees, books in the running brooks,
> Sermons in stones, and good in everything."

His vivacity was wonderful. His graceful and appropriate gestures added an indescribable charm to his conversation and lectures.

He possessed a noble simplicity of character. He was truthful and just. He hated evil speaking, and guarded against it with jealous care. He was a model physician. His presence in the sick room diffused sunshine and calm. He seemed to know by instinct the disease and the remedy. Dr. Brainard once said, that when he could think of no remedy, he sent for Dr. Blaney, and added, "he always has something to suggest."

He never became insensible to the sufferings of others. After watching for a lifetime the "tide of human woes," he was as tender as ever. His eyes would fill with tears at a story of suffering. He had not long ago for a patient a little girl, who had broken the upper third of her thigh, and was in danger of being crippled for life. He did for her all that skill could do and was completely successful. The restoration was complete. When the work was done and it was safe to remove her, his eyes filled with tears of joy, he took her in his arms and carried her to the carriage, drove her himself as far as she could safely go, and then brought her home in triumph.

His conversation was improving. Making no ostentatious display of knowledge, he brought from his storehouse "things new and old." He was gifted in anecdote and illustration. In his happy home, surrounded by his wife and children, he was genial and light-hearted, dispensing a graceful and refined hospitality.

In fine, he was a gentleman not of tinsel but of gold. He had sad hours, but on the whole a bright and happy life. Perhaps his sorrows are not to be deplored, for "sweet are the uses of adversity."

In his long illness, affection which never tired, watched beside him, and the Lord, as we trust, sent him the message of his love.

### MASONIC RECORD.

Dr. Blaney was widely known in the Masonic order in which he had been honored with high positions. He was a Past Master of Oriental Lodge, in Chicago, and Past Commander of Apollo Commandery Knights Templar; was the first Grand Commander of the Grand Commandery of Illinois; was Generalissimo, the third highest in rank of the officers of the Grand Encampment of the United States, and an honorary member, 33rd degree, of the highest, or Scotch rite, Masonic organization.

